# A New Site Preparation Protocol That Supports Bone Quality Evaluation and Provides Predictable Implant Insertion Torque

**DOI:** 10.3390/jcm9020494

**Published:** 2020-02-11

**Authors:** Stefan Velikov, Cristiano Susin, Peter Heuberger, Ainara Irastorza-Landa

**Affiliations:** 1 Nobel Biocare Services AG P.O. Box, CH-8058 Zürich-Flughafen, Switzerland; stefan.velikov@nobelbiocare.com (S.V.); peter.heuberger@nobelbiocare.com (P.H.); 2 Department of Periodontology, Adams School of Dentistry, University of North Carolina at Chapel Hill, Chapel Hill, NC 27599, USA; csusin@email.unc.edu

**Keywords:** osteotomy, site preparation, bone density, implant insertion torque, dental implants, bone quality

## Abstract

When preparing an implant site, clinicians often base their assessment of the bone on subjective tactile and visual cues. This assessment is used to plan the surgical procedure for site preparation, including how many drilling steps will be used. The subjective nature of bone evaluation, consequently, results in poor reproducibility and may lead to under or over preparation of the site. Recently, an unconventional site preparation protocol was developed in which the decision of which instruments to use is dictated by insertion torque of the novel site preparation instrument (OsseoShaper^™^, Nobel Biocare AB, Gothenburg, Sweden). The aim of this study was to quantify the correlation of the site preparation torques of the new instrument with bone density and maximum implant insertion torques. In vitro and in vivo data showed strong linear correlation between site preparation torque and density and resulted in reliable implant insertion torques, respectively. From our analysis, we conclude that this new instrument and protocol has the potential to eliminate the need for additional intraoperative bone evaluation and may reduce the risk of inadequate preparation of the site due to the ability to serve as a predictor of the final implant insertion torque.

## 1. Introduction

Successful dental implant placement depends on the appropriate assessment of the alveolar bone quality, which should inform the appropriate selection of the surgical technique, site preparation tools, and implant characteristics. Clinical examination in combination with radiographic assessment of the implant site provide clinicians with pre-operatory information essential for surgical planning [1,2,3]. Additionally, experienced clinicians adjust their surgical technique based on visual cues and tactile feedback during the implant site preparation [4,5,6,7]. Whereas the clinical and radiographic assessment of the implant site follow well-established parameters that can be promptly learned, the clinical judgment necessary to gauge bone characteristics has to be learned through experience [8,9]. In this regard, the clinical decision to under- or over-prepare the osteotomy has major consequences for primary implant stability and final implant position [10,11]. Although novel concepts, such as drilling energy [12] or intraoperative compressive tests [13,14], have been proposed to correlate with bone density and estimate bone quality, their application in dental clinics is not yet common practice.

**Figure 1:**
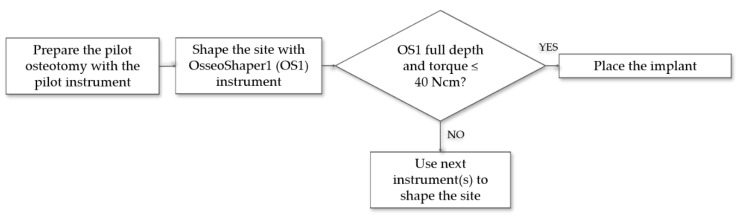
Simplified surgical procedure workflow.

Recently, a novel site preparation instrument was developed (OsseoShaper™, Nobel Biocare AB, Gothenburg, Sweden) featuring an innovative design consisting of a tapered body with an outer thread profile combined with a cutting flute and unconventional site preparation protocol. Following the pilot drill, the osteotomy is shaped with an implant-specific single use instrument, OsseoShaper1 (OS1), without irrigation at low speed (25–100 rpm) using a motorized drill unit with a maximum torque setting of 40 Ncm (Figure 1). The OS1 torque, measured by a drill unit with real-time torque display, determines the workflow for implant placement. If the OS1 achieves the desired depth and position with less than 40 Ncm, the implant can be placed. If the OS1 torque reaches 40 Ncm before it reaches the planned position, the next instrument should be used to further shape the implant site.

Preliminary observations of this new implant system and site preparation protocol indicated that the OS1 may enable clinicians to reliably assess bone quality and eventually predict final implant insertion torques. In this analysis of data previously generated from a series of in vitro and in vivo studies, we aimed to quantify the correlation of maximum OS1 torques generated during site preparation with bone density and maximum implant insertion torques of a dental implant (Nobel Biocare N1^TM^, Nobel Biocare AB, Gothenburg, Sweden).

## 2. Materials and Methods

The data presented in this work was analyzed from independently performed in vitro (bone surrogate and bovine bone) and in vivo (animal and human) studies between 24 October 2017 and 13 June 2019, sponsored by Nobel Biocare. In vitro and preclinical animal studies were performed as performance verification studies. Human data was extracted from an “ease of use” clinical handling questionnaire.

In vitro experiments were used to evaluate the correlation between the OS1 torques and bone density as well as implant insertion torque. Data from the preclinical animal study and clinical handling survey were used to evaluate clinicians’ estimation of bone quality compared to the protocol and to evaluate the correlation between the OS1 torques and implant insertion torque. Only implants that were placed immediately after OS1 were included (Figure 1). All site preparation protocols were performed according to the manufacturer’s instructions for use (IFU). For each dataset, inclusion and exclusion criteria were defined (Table 1) and are illustrated in Supplementary Figures S1–S4.

### 2.1. In Vitro Bone Surrogate

A total of seventy-two bone surrogates (Sawbones^®^, Malmoe, Sweden) of increasing densities (15 pcf (pound per cubic foot)–SKU #1522-02 (*n* = 24), 20 pcf–SKU #1522-03 (*n* = 24), 30 pcf–SKU #1522-04 (*n* = 24)) were prepared into ø8 × 40 mm cylinders. The pilot osteotomies were prepared using the pilot instrument (Supplementary Table S1) at 2000 rpm in a turning machine (TesT GmbH) to allow for reproducible and controlled initial site preparation. The depth of the osteotomies corresponded to the implant length (9, 11, or 13 mm). The sites were shaped using the corresponding OS1, followed by implant insertion to full depth (Supplementary Table S1). Both OS1 site preparation and implant insertion were performed at 30 rpm with a continuously controlled axial force in a turning machine (TesT GmbH). Implants in 15 pcf and 20 pcf densities were placed after the OS1, while 30 pcf bone surrogate required the next instrument in the protocol (Figure 1, Supplementary Figure S1). The maximum torques generated during site preparation and during implant placement were recorded with a torque sensor (TesT GmbH, T415.2Nm).

### 2.2. In Vitro Bovine Trabecular Bone

In a study performed at ARTORG Centre for Biomedical Engineering Research–University of Bern, 18 cylindrical trabecular bone samples (ø13.8 × 22 mm) were extracted from the tibiae plateau of dairy cows from a local slaughterhouse (Holzer Metzgerei, Hindelbank, Bern, Switzerland) using a diamond-coated hollow drill bit (Diamant Hohlbohrer Gesintert, 16 mm, Creative Glass MHS AG). The trabecular bone samples used in this study were homogeneous and did not contain any cortical layer. The bone samples underwent a micro-computed tomography (µCT) scan (µCT100, Scanco Medical, Brüttisellen, Switzerland) with a spatial resolution of 49.2 µm (energy: 70 kV, intensity: 200 mA, integration time: 90 ms). Samples were then segmented (threshold: 414 mgHA/ccm) to quantify the bone volume fraction (BV/TV) of each specimen. Afterwards, all samples were embedded into polymethylmethacrylate (PMMA) as previously described [7]. Bovine bone cylinders were frozen at −21° between uses.

The sites were prepared with either the pilot instrument (OS1 group) or with a conventional pilot drill (Conventional group) at 2000 rpm (Supplementary Table S2). All pilot osteotomies were prepared to a depth of 13 mm and a feeding rate of 1 mm/s in a computerized numerical control drilling platform (motor spindle: BFS-8015-12, Mechatron GmbH, Germany). Using a motorized drilling unit (Osseocare Pro; Nobel Biocare AB, Gothenburg, Sweden), sites of the OS1 group were further shaped using the OS1 at 75 rpm and those of the second group using a conventional drill at 2000 rpm with a manually controlled feeding rate. In all samples, implants were inserted to full depth and with a manually controlled feeding rate using the same drill unit at 25 rpm. The maximum torques generated during site preparation and during implant placement were recorded with a load cell (M-2025, Lorenz, Messtechnik GmbH, Germany) situated underneath the sample holder [7].

### 2.3. In Vivo Yucatan Minipig

In a preclinical study by the University of North Carolina at Chapel Hill, a total of fifty female or castrated male Mini Yucatan pigs, 18–24 months old, and with a weight range of 53–82 kg, underwent surgical extractions of mandibular premolars and first molar followed by a healing period of 12 ± 2 weeks to allow for the establishment of an edentulous alveolar ridge. Animal handling, pre-surgery procedures, surgical extractions, postsurgical procedures, and euthanasia were performed as previously reported [15].

During implant placement, a total of 134 implants were placed equicrestally at 25 rpm. During osteotomy preparation, clinicians were requested to estimate the bone quality according to Lekholm and Zarb [16]. Implant sites were prepared using a pilot instrument with maximum speeds not exceeding 2000 rpm and with irrigation, followed by the OS1 at 50 rpm without irrigation (Supplementary Table S3). Twenty-nine implants were placed immediately after OS1 at 25 rpm and have been considered for this evaluation (Supplementary Figure S3). Torques generated during site preparation and implant insertion were recorded with a surgical drill unit (Kavo MasterSurg^TM^,LUX, KaVo Dental GmbH, Biberach an der Riss, Germany) or with the manual wrench. Surgeries were performed at an accredited testing facility (AccelLab Inc) and the protocol was reviewed and approved by the Comité institutionnel de protection des animaux d’AccelLAB.

### 2.4. In Vivo Clinical Handling Surveys

Between 24 October 2017, and 13 June 2019, 123 patients received a total of 258 implants (Supplementary Table S4) in sites prepared using the OS concept. During the surgeries, clinicians’ observations and information on the implant placement procedure was collected in an “ease of use” handling survey. All surgeries were carried out in Europe with CE marked devices.

During the osteotomy preparation, clinicians estimated the bone quality according to Lekholm and Zarb [16]. Implant sites were prepared using a pilot instrument with maximum speeds not exceeding 2000 rpm and with irrigation, followed by the OS1 (9 to 14 mm, Supplementary Table S4) at speeds ranging from 25–100 rpm without irrigation. Two hundred and twenty-seven implants were considered for the evaluation of clinicians’ estimation and protocol used (Supplementary Figure S4). One hundred and forty-six implants were placed after OS1 at 25 rpm (Figure 1) and have been considered to evaluate the correlation between implantation torque and OS1 torque (Supplementary Figure S4). Torques generated during site preparation and implant insertion were recorded with clinicians’ surgical drill units or with the manual wrench.

### 2.5. Statistical Evaluation

Correlations were determined using linear regression analysis. Normality was checked with the Ryan–Joiner test. If data passed normality test, *t*-test or analysis of variance (ANOVA) 1-way tests were used. If the data did not pass the normality test, non-parametric tests, such as the Kruskal–Wallis and Mann–Whitney tests, were used. Significance was determined at a *p*-value of 0.05. Pearson correlations and Prediction Intervals were calculated using a linear regression model in Minitab 17.

## 3. Results

### 3.1. Correlation of OS1 Torque and Bone Density

**Figure 2:**
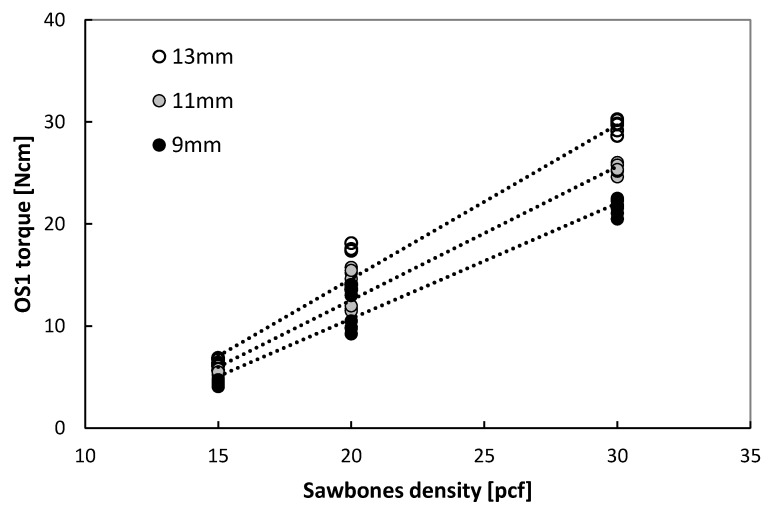
Linear regression of OS1 insertion torque in bone surrogate of different densities (pcf = pound per cubic foot).

The OS1 torques ranged from 4 to 30 Ncm in bone surrogate with densities of 15, 20, and 30 pcf (Figure 2, Supplementary Table S5). There was a positive strong linear correlation (*r* > 0.98 for all lengths, Supplementary Table S6) between the torque of the OS1 and bone surrogate density. The OS1 insertion torques were significantly different for each length per density (*p* < 0.001 for 15 pcf and 30 pcf and *p* = 0.01 for 20 pcf, Kruskal–Wallis).

**Figure 3:**
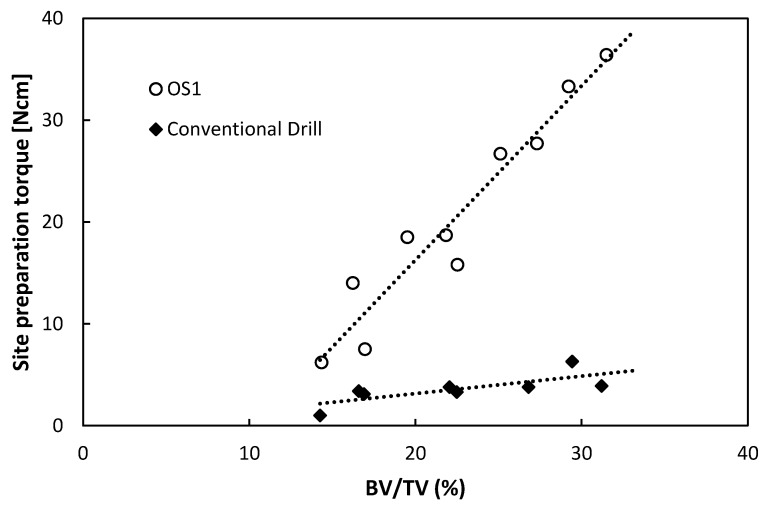
Linear regression of site preparation OS1 in bovine samples (*n* = 10) and of conventional drill (*n* = 8) with bone volume fraction (BV/TV) .

In bovine samples with a BV/TV between 14% and 31%, the OS1 torques ranged from 6 to 36 Ncm and the conventional drill torques from 1 Ncm to 6 Ncm. There was a strong positive linear correlation (*r* = 0.96, *p* < 0.001) between the OS1 torque and calculated bone density, whereas a weaker correlation (*r* = 0.75, *p* < 0.001) was observed between the insertion torque of conventional drill and BV/TV (Figure 3). Regression analysis evaluation showed lower predictability with a conventional drill than with OS1 in the evaluated range (R^2^(pred) = 0.021 versus 0.895 respectively, Supplementary Table S6).

### 3.2. Correlation of OS1 Torque and Implant Insertion Torque

**Figure 4:**
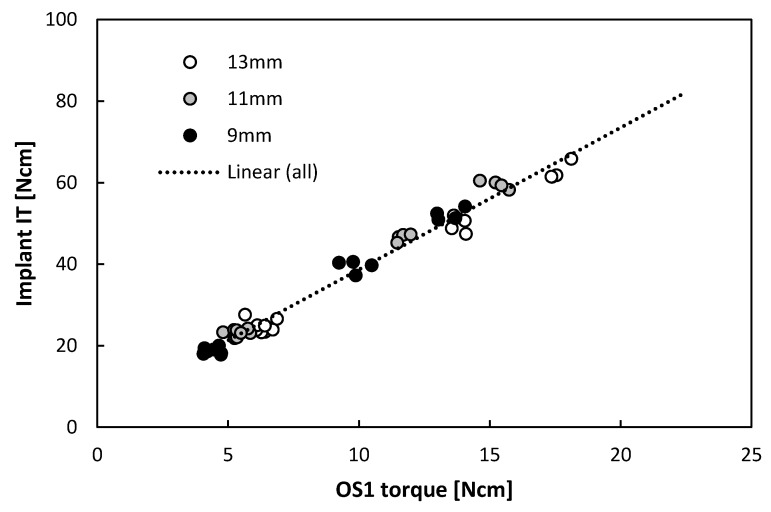
Liner regression of implant insertion torques versus OS1 insertion torques in 15 pcf and 20 pcf bone surrogate.

The implant torques ranged from 17 to 66 Ncm in bone surrogate with densities of 15 and 20 pcf (Supplementary Table S7) and a positive strong linear correlation (*r* = 0.99, *p* < 0.001) was found when pooling data from different lengths (Figure 4), despite their differences observed in Figure 2. The implant insertion torques were significantly different for each length in 15 pcf (*p* < 0.001, Kruskal–Wallis) but no significance could be found in 20 pcf (*p* = 0.056, Kruskal–Wallis). Regression analysis evaluation showed strong predictability of implant insertion based on OS1 torque in the evaluated range independently of the length (R^2^(pred) = 0.98, Supplementary Table S8).

**Figure 5:**
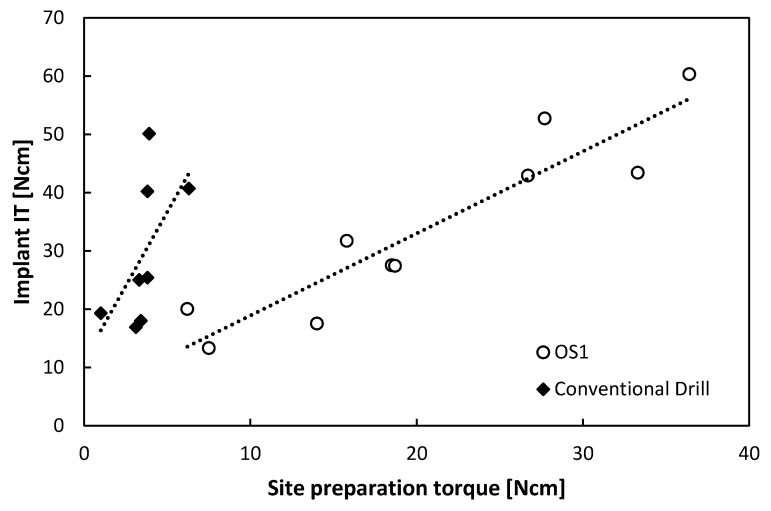
Linear regression of implant insertion torque versus OS1 insertion torque (*n* = 10) and versus conventional drill torque (*n* = 8) in bovine trabecular bone samples.

In bovine samples, the implant torques ranged from 13 to 60 Ncm (Figure 5). A positive strong linear correlation (*r* = 0.927, *p* < 0.001) was observed between the insertion torque of the OS1 and implant insertion torque in bovine trabecular bone (*n* = 10) and non-significant moderate positive correlation (*r* = 0.587, *p* = 0.126) for the conventional drill (*n* = 8). Indeed, contrary to the OS1 torque, no predictability of implant insertion based on a conventional drill torque can be expected (R^2^(pred) = 0.77 and R^2^(pred) = 0.06 respectively, Supplementary Table S8).

### 3.3. Clinicians’ Estimation of Bone Quality In Vivo

In minipigs, 29 implants were placed after the OS1 and the bone quality was assessed as Type IV or III in 65% (Table 2). However, in 35% of the cases, the bone was assessed as Type II, but the implants could be placed using only the OS1. For the 191 implants placed after the OS1 in human patients, the bone quality was assessed as Type IV or III in 75% (Table 2). However, in 25% of the cases, the bone was assessed as Type I or II.

### 3.4. Implant Insertion Torques In Vivo

**Figure 6:**
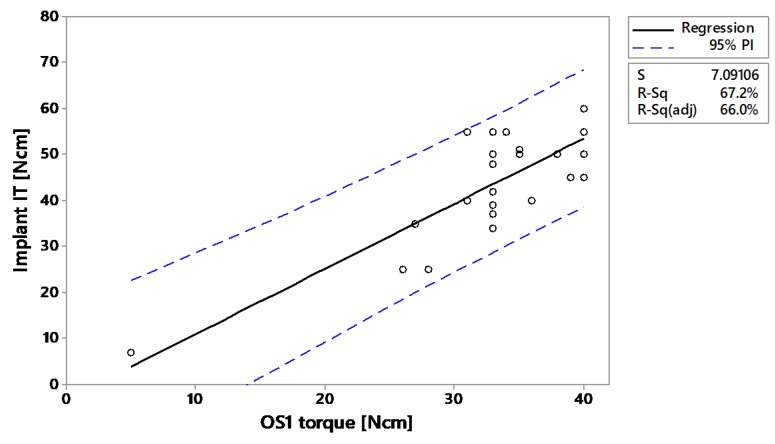
Linear regression of implant insertion torque versus OS1 torque in minipigs (*n* = 29).

**Figure 7:**
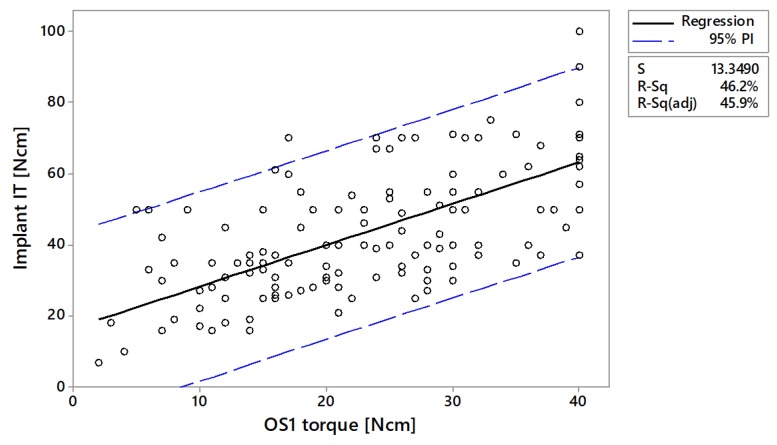
Linear regression of implant insertion torque versus OS1 torque in humans (*n* = 145).

In vivo, the linear correlation between OS1 torque and implant insertion torque was *r* = 0.82 (*p* < 0.001) for the minipigs and *r* = 0.68 (*p* < 0.001) in human subjects (Figure 6 and Figure 7). Predictability of implant insertion based on OS1 torque was R^2^(pred) = 0.623 based on minipig data and R^2^(pred) = 0.447 based on human data (Supplementary Table S9). In humans, the mean implant insertion torque was reported as 27.8 ± 13.5 Ncm when the OS1 torque was in the lower range of 0–10 Ncm (Table 3). According to the 95% prediction interval, the implant insertion torque could reach up to 54 Ncm. In the upper range of OS1 30–40 Ncm, the mean implant insertion torque doubles to 62.1 ± 14.9 Ncm with a 95% prediction of 25–90 Ncm. In minipigs, where a smaller implant was used (see Table S3 and Table S4), the averages and ranges were lower.

## 4. Discussion

Successful implant placement depends on the site anatomy, bone quantity, bone quality and proper surgical technique [17,18]. Although several instruments are available for site preparation, such as osteotomes, piezoelectric devices, ER:YAG laser or osseodensification burs, the most conventional surgical preparation technique uses successively increasing-diameter drills at high speed and require abundant irrigation [19]. In contrast, the newly developed OS1 instrument operates at low speed (<100 rpm) without the need for irrigation. Preclinical data suggest several biological benefits of this new site preparation concept, including increased cell viability at the site through elimination of high temperatures during site preparation and preservation of the granulated bone generated in situ [20].

Herein, we investigated if a novel site preparation instrument and protocol could be used to reliably assess bone quality and predict implant insertion torques in a variety of model systems and clinical practice. We observed a positive correlation between the OS1 torque and density and found that the implant insertion torque can be predicted from the OS1 torque in all evaluated scenarios. Ultimately, using this protocol may support clinicians’ decision-making during site preparation because the instrumentation sequence is dictated by the torques generated during site preparation.

Despite observing a moderate linear correlation between drilling torque and the BV/TV of bovine trabecular bone with a conventional protocol, the torques were too low across all bone qualities to serve as an indicator for bone density. This correlation can nevertheless be affected by operation conditions, such as rotational speed or feed rate [21]. In the in vitro studies, the rotational speed was set to 2000 rpm, maximum allowed speed in the IFU, to allow for a short test-duration, without investigating its impact. On the other hand, the broader range of OS1 torques across the densities serves as an indicator that this instrument responds to bone density in a manner that is more like how implant insertion torques behave. This difference arises from the design of the OS1 due to its outer thread profile (similar to an implant) that allows the OS1 to “pull” itself in and cut the bone at low speed. Consequently, the OS1 torque can be used as an indication for the encountered bone density and assist clinicians in their prediction of bone quality. In addition, the site preparation protocol results in highly predictable implant insertion torque in vitro: OS1 torque correlated strongly with the final insertion torque, both in bone surrogates and in trabecular bone, while the conventional drill torque was weakly correlated to the final implant insertion torque and was, hence, a weak predictor.

While in vitro bone density can be assessed objectively, clinicians rely on radiographs and their tactile sense to estimate bone quality. This estimation usually takes place during initial site preparation and relies on the resistance felt during drilling. Clinicians develop a good tactile sense over time and can learn to estimate the bone characteristics well. Therefore, it was not surprising, that when the OS1 was the last instrument before implant placement the clinicians assessed the bone in 65% (minipig) and 75% (human) of the cases as Type III or IV. Nevertheless, in the remaining 35% and 25% the initial estimation was Type I or II, and the bone quality was possibly overestimated. Using a conventional stepwise drill protocol this assessment may have led the clinician to use a protocol for dense bone which would have led to an overpreparation of the site. Instead, with the new site preparation protocol, the clinicians were guided to place the implants using only OS1.

Finally, the qualities of the OS1 site preparation protocol found in the in vitro experiments were also transferable to the in vivo cases. Although the correlation between OS1 torque and implantation torque is lower for the in vivo cases than in the in the in vitro experiments, to a certain extent, implant insertion torques can be predicted for a given OS1 torque.

The present analysis shows certain limitations. The different studies were performed independent of each other and the devices used differed between studies. However, the site preparation protocol was always based on the OsseoShaper^TM^ concept and all implants were based on the N1^TM^ concept. Also, implant insertion torque prediction was only possible when the OS1 was the last tool used. This is the case when the OS1 torque is less than 40 Ncm, which is expected to be true in soft to medium bone. This study did not investigate sites that required further preparation of the osteotomies. Regarding the models, one in vitro study was carried out in bone surrogates which is an often-used laboratory model for proof of concept implant testing [22,23,24] since it allows to perform the experiments under controlled parameters. Albeit bone surrogates do not fully replicate the mechanical properties of human bone, they are intended to provide consistent and uniform material with properties in the range of human cancellous bone [25]. The second in vitro study was performed in controlled laboratory conditions using trabecular bone samples from bovine tibia plateau [7]. The volume fraction of the trabecular bone across anatomic sites ranged between 14% and 31%, which would correspond to trabecular core found in posterior maxilla and anterior mandible, respectively [26]. All sites were prepared automated with a machine that allowed for very precise and reproducible drill depths and angulation. Consequently, the correlations found were very high. The minipig intra-oral model is known to represent a fully functional in-vivo anatomical model for dental implant placement and it has been increasingly used to assess the performance of dental implants [15,27,28]. In this study the encountered bone was rather dense and only 29 out of 134 osteotomies and implants were included in the analysis. This model may be better suited when evaluating implants placed in further enlarged sites instead of implants placed directly after OS1. Lastly, the clinical data were only a collection of surgery observations without predefined inclusion/exclusion criteria. Implants were placed in maxilla or mandible, in healed sites or extraction sockets, and using bone grafts in some cases but not in others. All these factors dilute the correlation between OS1 torque and implant insertion torque. Despite this, a correlation was found, and to a certain extent, implant insertion torque can be predicted based on the OS1 torque when its torque is below the 40 Ncm threshold (Table 3, Supplementary Table S9).

## 5. Conclusions

Within the limitations of this investigation, it was shown that the correlation between bone density and torque of the new site preparation instrument can assist in predicting bone quality and may serve as a predictor of the implant insertion torque. The novel site preparation protocol informs the procedural decisions and has the potential to reduce the risk of inadequate preparation of the site. Especially less experienced clinicians are expected to benefit from this guidance. Future clinical studies should prospectively evaluate our findings.

## Figures and Tables

**Table 1:** Summary of evaluations and inclusion/exclusion criteria.

Study Category	Evaluated Parameter	Inclusion	Exclusion
In vitro	1. Correlation between OS1 torque and density	All	None
	2. Correlation between OS1 torque and implant maximum insertion torque	Implants placed immediately after OS1	None
In vivo	1. Clinicians evaluation of bone quality	All	Data incomplete
	2. Correlation between OS1 torque and implant maximum insertion torque	Implants placed immediately after OS1	Data incomplete

**Table 2:** Estimated bone quality according to Lekholm and Zarb [16].

Estimated Bone Quality [16]	Minipig	Human
	*n* = 29	*n* = 191
Type I	*n* = 0 (0%)	*n* = 12 (6%)
Type II	*n* = 10 (35%)	*n* = 36 (19%)
Type III	*n* = 14 (48%)	*n* = 95 (50%)
Type IV	*n* = 5 (17%)	*n* = 48 (25%)

**Table 3:** Mean ± standard deviation (SD) and 95% prediction intervals (PI) of implant insertion torque for different OS1 torque ranges in minipigs and human subjects (Figure S5).

OS1 Torque Range [Ncm]	Minipig		Human	
	Implant IT Mean ± SD ^1^ [Ncm]	Implant IT 95% PI ^2^ [Ncm]	Implant IT Mean ± SD ^1^ [Ncm]	Implant IT 95% PI ^2^ [Ncm]
0–10	N/A (*n* = 1)	0–29	27.8 ± 13.5 (*n* = 17)	0–54
10–20	N/A (*n* = 0)	0–41	34.2 ± 11.5 (*n* = 44)	2–67
20–30	28.3 ± 5.8 (*n* = 3)	9–55	45.2 ± 13.7 (*n* = 46)	13–78
30–40	48.8 ± 7.7 (*n* = 25)	25–69	62.1 ± 14.9 (*n* = 39)	25–90

^1^ Mean and SD IT (= implant insertion torque) are calculated based on all data points within the specified range (Figure S5). ^2^ 95% PI is calculated based the lowest and highest values of the prediction lines within the specified range (Figure S5).

## Supplementary Materials

The following are available online at https://www.mdpi.com/2077-0383/9/2/494/s1, Figure S1: In vitro bone surrogate sampling with the corresponding inclusion/exclusion criteria, Figure S2: In vitro trabecular bovine tibia plateau sampling with the corresponding inclusion/exclusion criteria, Figure S3: Yucatan minipig sampling with the corresponding inclusion/exclusion criteria, Figure S4. Human data sampling with the corresponding inclusion/exclusion criteria. Table S1. Summary of in vitro bone surrogate material. Table S2. Summary of in-vitro bovine trabecular bone material. Table S3. Summary of in vivo minipig study material. Table S4. Summary of in vivo handling surveys’ material. Table S5: Mean ± Standard deviation for the maximum OS1 torques per length per density in bone surrogates. Table S6. Regression analysis of in vitro data: correlation between site preparation torque and density. Table S7. Mean ± Standard deviation for the maximum implantation torques per length per density in bone surrogates. Table S8. Regression analysis of in vitro data: correlation between implant insertion torque and site preparation torque. Table S9. Regression analysis of in vivo data: correlation between implant insertion torque and site preparation torque. Figure S5. Illustration of how mean ± SD obtained from all data in the shadowed area and 95% prediction intervals (PI) of implant insertion torque for a given OS1 torque ranges is calculated.
