# Supplementary material for: A New Site Preparation Protocol That Supports Bone Quality Evaluation and Provides Predictable Implant Insertion Torque
Source: J Clin Med. 2020 Feb 11;9(2):494. doi: 10.3390/jcm9020494 (PMC7074433; doi:10.3390/jcm9020494)
Supplement: Supplementary file 1 [file jcm-09-00494-s001.pdf]

Journal of
Clinical Medicine

MDPI

Supplementary Material

Appendix A - Illustration of the inclusion and exclusion criteria in each study

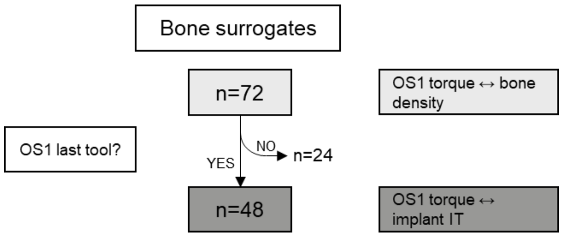

Figure S1. In-vitro bone surrogate sampling with the corresponding inclusion/exclusion criteria

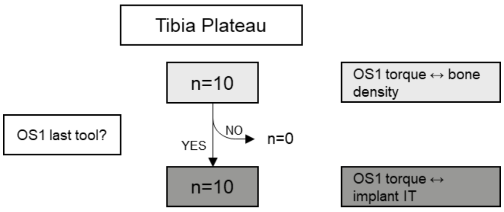

Figure S2. In-vitro trabecular bovine tibia plateau sampling with the corresponding inclusion/exclusion criteria

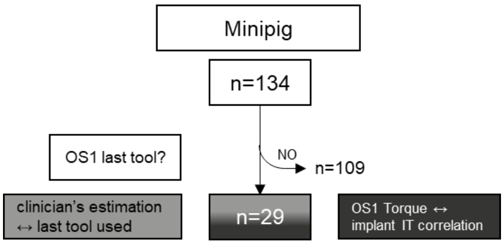

Figure S3. Yucatan minig sampling with the corresponding inclusion/exclusion criteria

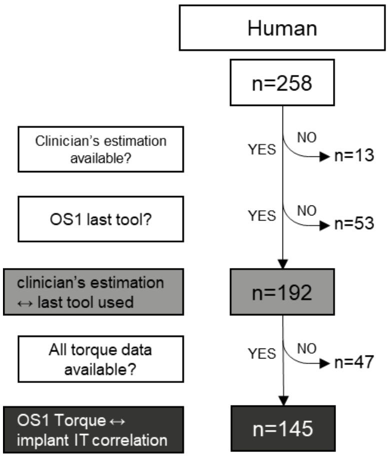

Figure S4. Human data sampling with the corresponding inclusion/exclusion criteria

Appendix B – Material used in different studies

Table S1. Summary of in-vitro bone surrogate material

Category	Description	Article number	n	Speed
Pilot instrument	Ø1.8/2.4 x 8-14mm	300561	72	2,000rpm
OS1 concept instrument	RP 4.0 mm x 9mm	300027	24	30rpm
	RP 4.0 mm x 11mm	300028	24	30rpm
	RP 4.0 mm x 13mm	300029	24	30rpm
Implant	N1 TiUltra TCC RP 4.0 x 9mm	300014	16	30rpm
	N1 TiUltra TCC RP 4.0 x 11mm	300015	16	30rpm
	N1TiUltra TCC RP 4.0 x 13mm	300016	16	30rpm

Table S2. Summary of in-vitro bovine trabecular bone material

Category	Description	Article number	n	Speed
Pilot instrument	∅1.8/2.4 x 8-14mm	300561	8	2,000
OS1 concept instrument	RP 4.0 mm x 13mm	300029	10	75
Conventional pilot drill	Twist Drill with Tip Ø 2 x 7-15 mm	32297	8	2,000
Conventional drill	TwistStep Drill Ø2.4/2.8 x 10-18mm	32262	8	2,000
Implant	N1 TiUltra TCC RP 4.0 - 13mm	300016	8	25

Table S3. Summary of in-vivo minipig study material

Category	Description	Article number	n	Speed
Pilot instrument	∅1.8/2.4 x 8-14mm	300561	N/A	≤2,000
OS1 concept instrument	NP 3.5 - 11mm	300371	25	50
	NP 3.5 - 11mm	300882	4	50
Implant	N1 TiUltra TCC NP 3.5 -11mm	300378	25	25
	N1 TiUltra TCC NP 3.5 -11mm	300858	4	25

Table S4. Summary of in-vivo handling surveys's material

Category	Description	Article number	n	Speed
Pilot instrument	Precision Drill	36118	N/A	≤2,000rpm
	1.8/2.4 x 8-14mm	300561		
OS1 concept instrument	RP 4.0 mm x 9mm	300027	87	25-50rpm
	RP 4.0 mm x 11mm	300028		
	RP 4.0 mm x 13mm	300029		
	RP 4.0 mm x 10mm	300154	104	25-50rpm
	RP 4.0 mm x 12mm	300155		
	RP 4.0 mm x 14mm	300156		
Implant	N1 TiUltra TCC 4.0 - 9mm	300014	87	25rpm
	N1 TiUltra TCC 4.0 - 11mm	300015		
	N1 TiUltra TCC 4.0 - 13mm	300016		
	N1 TiUnite CC 4.0 -10mm	300150	104	25rpm
	N1 TiUnite CC 4.0 -12mm	300151		
	N1 TiUnite CC 4.0 -14mm	300152		

Appendix C - Supplementary results data

Table S5. Mean±Standard deviation for the maximum OS1 torques per length per density in bone surrogates

	15 pcf	20 pcf	30 pcf
9 mm	4.45±0.27	11.65±1.85	21.72±0.64
11 mm	5.38±0.31	13.46±1.83	25.33±0.39
13 mm	6.32±0.36	15.72±1.91	29.38±0.66

Table S6. Regression analysis of in-vitro data: correlation between site preparation torque and density

		Pearson correlation		Linear regression				
		r	p-value	Equation (y=ax+b)(1)	R^2	S(2)	R^2(pred)(3)	p-value
Figure 2	OS1 9 mm	0.983	<0.001	y = 1.311x - 11.89	0.966	1.38	0.962	<0.001
	OS1 11 mm	0.988	<0.001	y = 1.310x - 13.65	0.976	1.33	0.973	<0.001
	OS1 13 mm	0.989	<0.001	y = 1.513x - 15.65	0.979	1.49	0.975	<0.001
Figure 3	OS1	0.963	<0.001	y = 1.711x - 17.94	0.927	2.94	0.895	<0.001
	Conventional drill	0.748	0.033	y = 0.1717x – 0.282	0.559	1.04	0.021	0.033

(1)y= site preparation torque in Ncm (OS1 or Conventional drill), x= density (pcf or BV/TV(%))

(2)S: Standard Error of the Regression

(3)R^2(pred): it determines how well the model predicts new observations based on the linear regression equation

Table S7. Mean±Standard deviation for the maximum implantation torques per length per density in bone surrogates

	15 pcf	20 pcf
9 mm	18.70±0.74	45.81±0.74
11 mm	23.13±0.85	53.03±6.96
13 mm	24.79±1.57	56.15±7.18

Table S8. Regression analysis of in-vitro data: correlation between implant insertion torque and site preparation torque

		Pearson correlation		Linear regression				
		r	p-value	Equation (y=ax+b)(1)	R^2	S(2)	R^2(pred)(3)	p-value
Figure 4	All length pooled	0.99	<0.001	y = 3.478x + 3.904	0.979	2.33	0.979	<0.001
Figure 5	OS1	0.927	<0.001	y = 1.408x + 4.83	0.860	6.21	0.768	<0.001
	Conventional drill	0.587	0.126	y = 5.08x + 11.3	0.344	10.95	0.057	0.126

(1)y= maximum implantation torque (Ncm), x= site preparation torque in Ncm (OS1 or Conventional drill)

(2)S: Standard Error of the Regression

(3)R^2(pred): it determines how well the model predicts new observations based on the linear regression equation

Table S9. Regression analysis of in-vivo data: correlation between implant insertion torque and site preparation torque

		Pearson correlation		Linear regression				
		**r**	**p-value**	**Equation (y=ax+b)** (1)	**R^2**	**S**(2)	**R^2(pred)**(3)	**p-value**
Figure 6	Minipig	0.82	<0.001	y = 1.416x -3.158	0.672	7.09	0.623	<0.001
Figure 7	Human	0.68	<0.001	y = 1.165x+ 16.61	0.462	13.35	0.447	<0.001

(1)y= maximum implantation torque (Ncm), x= OS1 torque in Ncm

(2)S: Standard Error of the Regression

(3)R^2(pred): it determines how well the model predicts new observations based on the linear regression equation

Appendix D - Supplementary illustration for prediction interval values

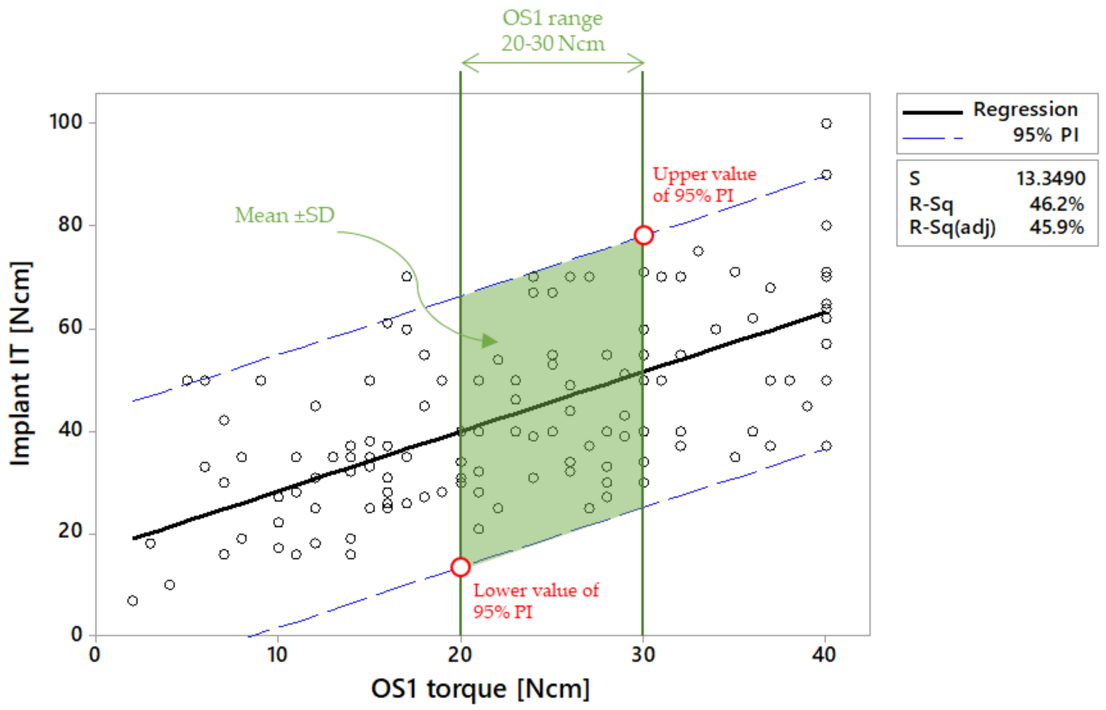

Figure S5. Illustration of how mean±SD obtained from all data in the shadowed area and 95% prediction intervals (PI) of implant insertion torque for a given OS1 torque ranges is calculated
